# Post-Operative Capsular Opacification: A Review

**Published:** 2007-12

**Authors:** Shetal M. Raj, Abhay R. Vasavada, S. R. Kaid Johar, Vaishali A. Vasavada, Viraj A. Vasavada

**Affiliations:** *Iladevi Cataract and IOL Research Centre, Raghudeep Eye Clinic, India*

**Keywords:** posterior capsule opacification, capsular opacification, cataract, phacoemulsification, crystalline lens

## Abstract

Post-operative capsular opacification is a multifactorial physiological consequence of cataract surgery. Opacification involving the central posterior capsule has a significant impact on high and low contrast acuity and low contrast sensitivity. The assessment of Posterior Capsule Opacification (PCO) on cadaver eyes, experimental studies, culture models and in clinical studies has provided an understanding of its pathogenesis. The proliferation, migration and abnormal differentiation of residual lens epithelial cells and fibers in the capsular bag have been implicated in the pathogenesis of PCO. The incidence and severity of PCO correlates to the meticulous use of surgical techniques, IOL optic edge designs and IOL materials. This article summarizes the clinical studies with recommendations for retarding the development of central PCO. It discusses experiments with pharmacological agents broadly categorized as anti-inflammatory, immuno-modulating, antiproliferative, antiadhering, antitransdifferentiating agents for the prevention of PCO. These studies will remain critical for future endeavors undertaken for eradication of PCO.

## INTRODUCTION

**Figure 1:**
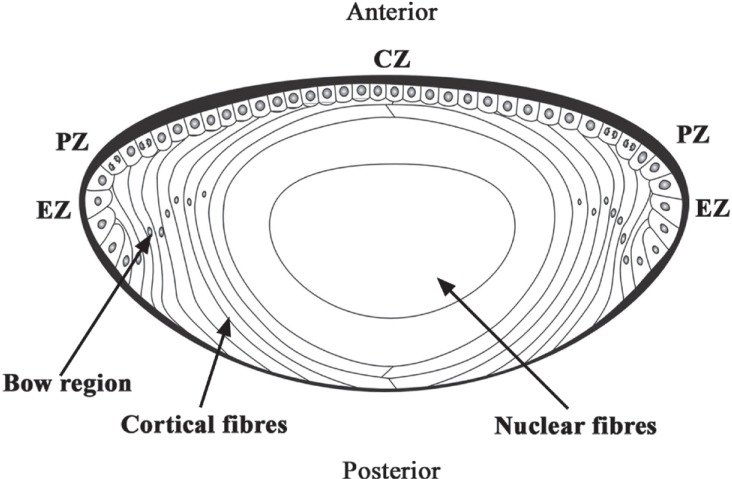
The anatomy of the human crystalline lens.

Cataract is an opacification of the transparent crystalline lens. The lens is a biconvex, elliptical, semi-solid, avascular body. It is enveloped by a thick capsule, which is a basement membrane of the lens epithelium. The lens epithelial cells (LECs) line the interior surface of the anterior, pre-equatorial, and equatorial regions of the lens capsule (Figure 1). In the equatorial region of the lens, the LECs undergo terminal differentiation to form lens fibers that are laid down in a concentric manner. From the lens capsule to the center of the lens, these concentric layers of lens fibers are identified broadly as cortex and nucleus. The nucleus contains the oldest lens fibers while the newer ones lie superficially in the cortex. Generally, cataract is associated with old age; however, it may be present in neonates or may develop at any time in the lifespan of an individual. The most common risk factor of cataract is aging. Other risk factors are exposure to ultraviolet (UV) light, inadequate nutrition, cigarette smoking, high alcohol intake, diabetes, and long-term use of antipsychotic medications or steroids (1). Epidemiological studies have emphasized a relationship between sunlight, particularly long wavelength UV (300-400 nm) and UV B (290-320 nm), and the formation of cataracts (2, 3). Cataract leads to dimness of vision while focusing either on distant or near objects and corrective glasses may or may not help rectify it. The only viable option for treating cataracts is to extract it surgically and replace it with an artificial intraocular lens (IOL).

Previously in intracapsular cataract extraction, the whole lens along with the entire capsule was extracted. In this method, the LECs are completely removed but there is no capsule left for IOL implantation. In the modern approach to extracapsular cataract extraction, the surgeon extracts all the lens fibers but leaves behind an intact posterior capsule and peripheral anterior capsule of the capsular bag for IOL implantation. Along with the residual capsule, LECs that have the potential to lay down cellular products are also left behind. These again cause opacification, which is referred to as capsular opacification. Thus, capsular opacification is a physiological postoperative consequence of an uneventful uncomplicated extracapsular cataract surgery. Capsular opacification is different from the intraoperative opacification that takes place in the intact lenses. It is known as a plaque which can either be in the anterior and/or posterior capsule. Plaques are frequently associated with white mature cataract (4).

## CLASSIFICATION

**Figure 2:**
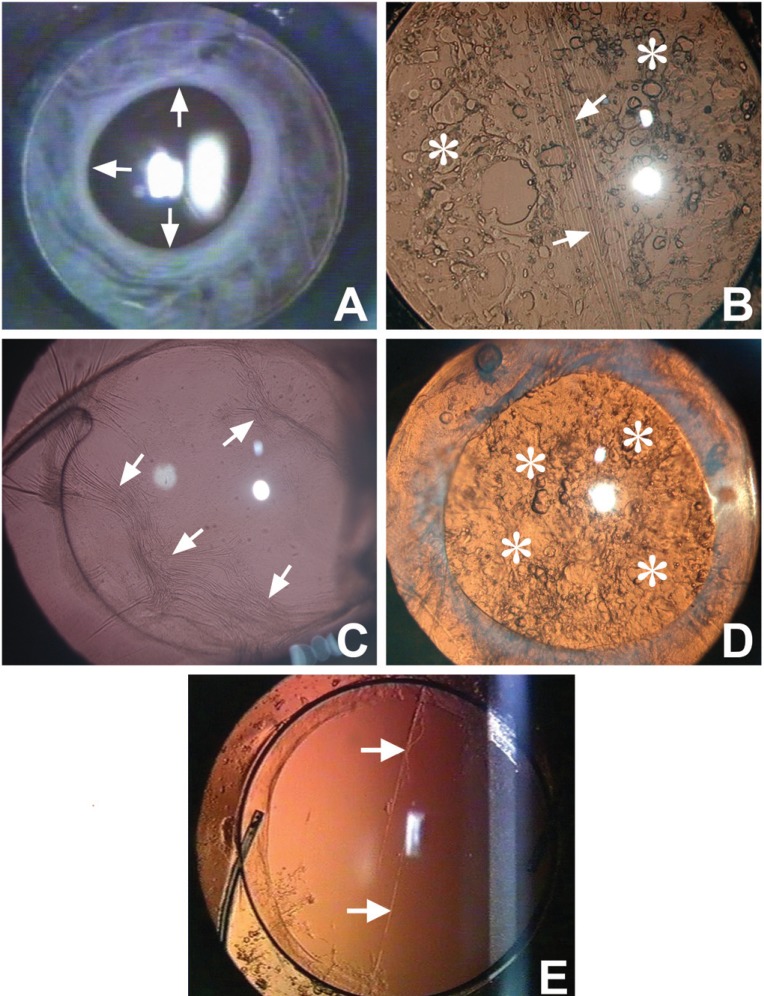
Eyes showing various forms of capsular opacification. **A,** Extensive anterior capsule opacification; **B,** Mixed type of PCO with fibrous (arrow) and pearl type areas (asterisk); **C,** Fibrous form of posterior capsule opacification (arrow); **D,** Proliferative or pearl form of posterior capsule opacification (asterisk); **E,** Linear posterior capsule opacification.

Postoperative capsular opacification can be classified based on its location into anterior and posterior capsule opacification (Figure 2A-2E).

### Anterior Capsule Opacification (ACO)

Opacification of the residual peripheral anterior capsule is known as anterior capsule opacification (Figure 2A). This is pronounced in eyes in which a continuous curvilinear capsular opening overlaps the IOL edge and peripheral anterior IOL surface (5, 6). This opacification sets in by the first postoperative month and continues until 6 months (7). Some studies have cited the IOL material and the IOL design as influencing factors for ACO (8, 9). An analysis of the opacified anterior capsule lying over the IOL can provide inference on the chemical and physical properties of the different IOL materials. Increased capsulorhexis contraction has been noted following implantation of silicone IOLs with sharp optic edges (8) or a plate haptic silicone design. It has been observed to be the lowest with acrylic IOLs (10). The incidence of ACO and contraction is high in eyes with retinitis pigmentosa (11) and in diabetic patients (12). Werner *et al* have clinically graded ACO from 0 to IV (10). A histopathological examination of the opacified anterior capsule of rabbits showed fibroblast-like cells, transformed from LECs (13).

The increased capsulorhexis contraction could be a hindrance, especially during examination of the peripheral fundus. Reducing ACO is especially important because it can cause decentration of the IOL. With multifocal IOLs being used, it is of paramount importance to maintain good centration to achieve a good visual outcome (14). Aspiration of LECs from the anterior capsule during cataract surgery has shown to reduce the capsulorhexis aperture contraction 3 months after cataract surgery (15). In our clinical trial, scraping of the anterior capsule to aspirate the LECs was not mandatory when considering implantation of the AcrySof IOL (16).

### Posterior Capsular Opacification (PCO)

Posterior capsular opacification, referred to as ‘secondary cataract’ or ‘after cataract’, develops over the clear posterior capsule a few months to a few years after an uneventful cataract surgery (Figure 2A, 2B, 2C). PCO results from the growth and abnormal proliferation of LECs on the capsule at the time of cataract surgery. These cells migrate to the posterior capsule where they approach the central visual axis and cause visual axis obscuration, resulting in dimness of vision.

The PCO has two forms, fibrous and pearl. Sometimes a combination of both is also found (Figure 2B). The LECs that line the anterior capsule are believed to be responsible for fibrous PCO (Figure 2C). Clinically it is seen as a wrinkling on the posterior capsule at the site of fusion of the anterior and posterior capsules. A histological examination of the fibrous PCO shows extracellular matrix (ECM) accumulation and the presence of elongated fibroblast cells positive to vimentin and alpha-smooth muscle actin (17). The LECs lining the pre-equatorial zone are responsible for the pearl or proliferative PCO (Figure 2D). On examination it shows clusters of swollen, opacified differentiated LECs called bladder or Wedl cells (18).

### Other forms

The interlenticular opacification develops after cataract surgery between 2 intraocular lenses (IOLs) placed in the capsular bag (19). Linear posterior capsule opacification along persistent striae has also been noted (Figure 2E). The striae appear to create a channel that allows epithelial cells to bypass the barrier created by the square-edge design of the IOL and migrate posteriorly in a linear pattern (20, 21). These can cause visual difficulties in some patients.

## INCIDENCE AND IMPACT

The incidence of PCO is known to range from as high as 50% to as low as <5% in eyes undergoing cataract surgery for uncomplicated senile cataracts (22-24). PCO reduces visual acuity when the central area (inside the pupillary aperture) is involved (25). PCO within the central 3 mm zone of the posterior capsule affects high contrast sensitivity, low contrast acuity, and sensitivity psychophysical test results with differing degrees of sensitivity. Forward light-scatter is the most sensitive, followed by contrast sensitivity and visual acuity (26). The amount of PCO and the size of the area analyzed are relevant since this could interfere with the appropriate interpretation of findings on scanning with a laser polarimetry (27).

## ASSESSMENT AND ANALYSIS

Different methodologies have been used in experimental and clinical studies.

### Human Autopsy Studies

An examination of human autopsy eyes from the posterior (Miyake-Apple) view, complemented by microscopic analyses is useful in evaluation of IOL-capsular bag interaction, role of the surgical technique, and evaluation of new IOL designs (28, 29) in retarding development of central PCO.

### Experimental Studies and Culture Models

Animal studies and *in vitro* models have been used to assess the mechanism of PCO formation (30, 31). The development of PCO is faster in rabbits and has been found to be suitable for studies to explain human PCO development (32). In *in vitro* models, the LECs are cultured either in a medium or in capsular bags prepared from porcine eyes (33) or human cadaver eyes (34). Culture of animal LECs (35, 36), human LECs, and human lens epithelial cell lines (37, 38) have been used to study the mechanisms involved in the pathogenesis of PCO. Culture studies can also detect potential therapeutic targets in the treatment of PCO. A digital quantification of the cell migration onto the posterior capsule and capsule contraction can also be done in these models (39).

### Clinical Studies

In clinical studies, PCO can be assessed by either comparing the presence or absence of PCO within the central visual axis or comparing the Nd: YAG capsulotomy rates (40, 41). PCO-induced loss of contrast sensitivity has also been used as a method for clinical studies on PCO (42). However, these are subjective comparisons, which rely on the patients’ perceptions of the difficulties involved and need longer follow-up before a conclusion can be arrived at. Another option is to assess PCO on slit lamp-acquired retro-illumination images after complete mydriasis. This is however, subjectively graded based on the severity of PCO. The uneven illumination and low resolution of the images obtained with a slit lamp has led to the increasing use of digital images.

Digitally acquired retro-illumination photography allows quick acquisition of PCO images. It provides excellent image quality and high reproducibility. This technique forms a sound basis for subjective grading of the intensity of PCO (score 0 to 4) (43) as well as for automated quantification of PCO with new software systems. These systems can detect early progression of PCO as well as visually significant PCO. Images with no clinical PCO produce very low scores in the analysis (44). Several software systems are available for analysis of PCO on digitally acquired retroilluminated images such as Evaluation of Posterior Capsule Opacification (EPCO) (45) and the Automated Quantification of After-Cataract (AQUA) automated PCO analysis program (46).

### Analysis

In the POCOman software, images are analyzed by a set protocol of defining the area of the posterior capsule, removing the Purkinje light reflexes by intensity segmentation, contrast enhancement, filtering to enhance low-density PCO, and variance analysis using a co-occurrence matrix to assess the texture. It provides a semi objective assessment of PCO and is valid and repeatable (47, 48). The POCOman software determines the percentage area of PCO and assigns a severity score. The results of the POCOman system have correlated well with the results of the automated POCO system as well as the results of the clinical evaluation.

These automated image-analysis systems provide an objective PCO assessment and are valuable tools for clinical studies evaluating the development and prevention of PCO. The OSCA is a new system of PCO analysis that incorporates flash detection and removal, registration and subsequent merger of images for artifact removal, as well as location sensitive texture analysis. The system is referred to as the open-access systematic capsule assessment (OSCA) (49).

Another system that captures high resolution images is the EAS-1000 system (Scheimpflug videophotography) (50). However, the IOL material has been seen to significantly influence the scatter light density measurements and thus the intensity of PCO quantified by this system cannot be directly compared with different optic materials. This makes the system less applicable in evaluating PCO (51). The optical coherence tomography (OCT-1) has been used to quantify PCO and to discriminate between different types of PCO (52). PCO evaluation with OCT is based on peak intensity (PI) and posterior capsule thickening (PCT), with PCT indicating the distance between two reflectivity spikes with an approximate axial resolution of 10 μm. Ultra High-Resolution (UHR) OCT images using 1.4 μm axial × 3.0 μm transverse resolutions have corresponded to the histology sections (53). Using a wave-length of 800 nm, the anterior and posterior capsules, area of growth of the LECs, ECM production, and IOL can be clearly visualized. The extent of the capsular bag adhesion to the IOL and the amount of PCO can be detected. The improved resolution can make UHR OCT a powerful tool in anterior segment imaging, to evaluate the capacity of IOL materials and models to induce capsular bag adhesion and to determine the area of origin of PCO after cataract surgery.

## PATHOGENESIS

The development of PCO is a very dynamic process and involves three basic phenomena: proliferation, migration, and differentiation of residual LECs.

### Proliferation

The proliferation of residual LECs is highest in the 3 to 4 days after surgery (54). The precise reason for the response of LECs, which leads to their proliferation and the subsequent PCO development, is not known. The removal of lens fiber mass during cataract surgery seems to alter the local environment, inducing proliferation of LECs (55). Residual cortex may also promote proliferation of PCO. Besides LECs, melanocytes from the iris and cells released from the blood due to the breakdown of the blood aqueous barrier may also contribute to the initial proliferation of cells (56). This inflammation response may be exacerbated by the presence of a foreign material, that is, the IOL. The response of the LECs in the lens capsular bag is executed by the autocrine or paracrine systems (57). In the autocrine system, the residual LECs themselves secrete various cytokines that control the development of PCO (58). The evidence of the autocrine system is based on the observation that LECs in culture maintain themselves in a protein-free medium. A variety of cytokines including fibroblast growth factor (FGF), platelets derived growth factor (PDGF), hepatocyte growth factor (HGF), epidermal growth factor (EGF), insulin like growth factor (IGF), transforming growth factor β (TGFβ), interleukin 1 and 6, have shown to regulate the behavior of LECs *in vitro*, but little is known about their *in vivo* effects that lead to PCO (57). Basic FGF (bFGF) in the aqueous humor of rabbits increases after surgery and stimulates proliferation of LECs (59). TGFβ concentration in rabbit aqueous humor decreases after surgery and returns to the preoperative level after about 30 days (59). TGFβ induces epithelial mesenchymal transition (EMT) of LECs and leads to production of extracellular matrix (60). It seems that the initial fall in TGFβ allows proliferation of LECs due to the action of bFGF and other growth factors (57, 61). In children, the higher density of the LECs and greater number of mitotically active cells result in a higher growth potential (62, 63).

### Migration

The migration of LECs toward the posterior capsule and their subsequent attachment to the posterior capsule are facilitated by various cell attachment molecules present on the LECs. These molecules include various integrin subunits (64, 65), cell adhesion molecules (CAM) (66) and hyaluronan receptor CD 44 (67). Gly-Arg-Gly-Asp-Ser-Pro (GRGDSP) RGD peptide inhibited cell attachment, and migration on laminin and fibronectin that have Arg-Gly-Asp (RGD) peptide sequences (38). Matrix metalloproteinases (MMPs), which are a group of proteolytic enzymes, are essential for cell migration and cell mediated contraction following wound healing (39).

### Differentiation

The LECs have the ability to undergo both normal and abnormal differentiation. The normal pre-planned differentiation of LECs leads to the formation of pearl-like structures in the posterior capsule known as bladder cells (40). The bladder cells are quite dynamic, show reorganization and even disappear within a time period (68). They have a homogenously granular cytoplasm with pyknotic or no nuclei and do not express αSMA (69). The LECs of the equatorial zone are believed to have a stronger tendency to form these cells compared with the anterior LECs.

The abnormal differentiation of LECs takes place in a form of EMT. TGFbeta induces the EMT of LECs and FGF promotes the survival of TGFbeta-affected cells (70). The EMT of LECs leads to the formation of myofibroblast cells that are positive to αSMA. The formation of myofibroblast also takes place locally at the cut margin of the anterior capsule (71). The appearance of αSMA gives a retractile property to the cell, causes wrinkling of the posterior capsule, and forms fibrous PCO. The myofibroblasts also secrete various types of ECM proteins such as fibronectin, collagen type I, collagen type III, chondroitin sulphate, dermatan sulphate and keratan sulphate (72, 73) amongst others.

## RISK FACTORS

Several systemic and ocular associations have been cited for influencing the development of PCO. The review of case records to evaluate the risk factors for PCO has revealed no correlation between PCO and age, gender, or axial length. At the one- year follow-up, diabetic patients had significantly severe PCO after cataract surgery when compared with non-diabetic patients (74). However, amongst the diabetics, the stage of diabetic retinopathy and the systemic status of diabetes did not seem to correlate with the degree of PCO (75). Myopic eyes were postulated to have an increased risk of PCO probably because IOL implantation was deferred in them. However study with IOL implantation in myopic eyes showed no association between degrees of myopia to degree of PCO. The incidence of PCO is also high in eyes with uveitis (77). In these eyes, hydrophobic acrylic IOLs have shown to provide a better visual outcome and lower incidence of PCO than silicone, PMMA, or heparin-surface-modified PMMA IOLs (78). Patients with myotonic dystrophy have required multiple capsulotomies following cataract surgery (79). Similarly, patients with retinitis pigmentosa showed a significantly higher incidence and density of PCO (80). In traumatic cataracts, the incidence of PCO is significantly higher and has been quoted to be as high as 92% at the three-year follow-up (81).

## PREVENTION

### Surgical Techniques

**Continuous Curvilinear Capsulorhexis.** In aphakic eyes, the creation of a continuous curvilinear capsulorhexis was found to delay the development of central visual obscuration. In such eyes the fusion of the edge of the continuous curvilinear capsulorhexis to the posterior capsule forms a Soemmering’s ring. This ring provides a closed environment, which restricts the migration of the LECs towards the central posterior capsule. The ring contains residual LECs, residual cortical fibers, and differentiated LECs. Excessive proliferation of residual LECs or migration of LECs across this ring has been reported at longer follow-ups. Because the residual LECs are the main culprits, their thorough removal can prolong the development of a central PCO (18).

**Cortical Cleaving Hydrodissection.** Cortical cleaving hydrodissection causes a cleavage between the lens capsule and the cortex (82). In a study done on cadaver eyes, Peng and coauthors reported that the hydraulic force exerted by cortical cleaving hydrodissection could remove the LECs (83). Our study has also suggested that if cortical cleaving hydrodissection could be done in multiple quadrants, the time required to aspirate the epinucleus and cortex would be shorter, and the removal complete (84). High-resolution digital retro-illumination images of the posterior capsule in these eyes at 4 years revealed no difference in the incidence of PCO, but the percentage area of the central posterior capsule affected by PCO was significantly lower in eyes that had multi-quadrant cortical cleaving hydrodissection than in those that did not (85).

**Hydrodissection Combined with Rotation.** In an experimental laboratory study of fresh human cadaver eyes, cortical cleaving hydrodissection combined with rotation removed significant quantities of LECs and residual cortical fibers by way of friction (86). The fact that loose LEC’s were found in the fluid collected from capsular bag confirms that these two procedures effectively scrap the LECs that otherwise remain attached to the capsule (87).

**Cortical Clean-up.** Bimanual irrigation and aspiration for cortical clean-up facilitates access to the deep fornices of the capsular bag, especially in the sub-incisional quadrants (85, 86). The thorough removal of residual cortical fibers reduces the number of mitotically active cells that have the potential to proliferate and migrate across the central visual axis (88).

**In-the-bag IOL Fixation.** In-the-bag fixation of the optic and the haptic is required to consistently reduce the incidence of central PCO. Tan and associates noticed an increased incidence of fibrosis type PCO in cases of ciliary sulcus fixation (89). In-the-bag fixation is facilitated by creating a continuous curvilinear capsulorhexis. It functions primarily to enhance the IOL optic barrier effect (88). In-the-bag fixation, along with other measures related to surgical techniques and IOL choice, have reduced the incidence of Nd: YAG laser posterior capsulotomy to 0.9% with Alcon AcrySof IOL (22).

**Anterior Capsule Overlap of IOL Optic.** When the size of the anterior capsulorhexis is larger than that of the IOL, there is an increased incidence of fibrous PCO since the anterior epithelium remains apposed to the posterior capsule. It is believed that with a capsulorhexis smaller than the IOL optic the adhesion between the anterior capsule and the IOL optic keeps the anterior lens epithelium away from the posterior capsule. This would decrease the incidence of migration of the anterior LECs behind the IOL optic (89). On the other hand it has also been postulated that a capsulorhexis larger than the IOL optic allows adhesion of the anterior and posterior capsules, forming a Soemmering’s ring. This could contain the cells and regenerative cortical matter, preventing LEC migration onto the visual axis (90, 91). The size of this continuous capsulorhexis opening has not shown any correlation with the degree or severity of PCO (92).

There are several probable mechanisms of anterior capsule overlap over the IOL optic that help in delaying development of central PCO, including the “shrink wrap” effect (90), the concept of the barrier effect of an IOL optic edge (22), the creation of a discontinuous capsular bend by an IOL with a sharp optic edge (93), and the bioactivity based Sandwich theory (94).

Different IOL materials show different PCO rates due to the variations in the overlap of the anterior capsule with the IOL optic (95, 96). The total cover of the IOL optic remains an important factor, especially in eyes implanted with PMMA IOLs (95) and those with silicone IOLs (97). However, with the AcrySof IOL implantation, the size of the rhexis may not be a crucial factor in the development of a central PCO (96). Capsular tension rings have shown to reduce the formation of capsular opacification (98).

**Buttonholing of PC.** The potential of primary posterior continuous curvilinear capsulorhexis (PCCC) to prevent PCO 2 years after surgery has shown encouraging results (99). Posterior optic buttonholing through a PCCC has also shown to preclude lens epithelial cells from accessing the retrolental space. The sandwiched posterior capsule blocks optic contact and thus, prevents fibrosis of the anterior capsule (100). This technique is skill dependant and could be potentially demanding in eyes with an increased risk of posterior segment complications.

**Bag-in-the-lens implantation.** The culture of postmortem capsular bags and *in vivo* animal models have used a Bag-in-the-lens implantation technique to limit LEC proliferation. In this technique, anterior and posterior capsule flaps of similar sizes are inserted in a flange of the IOL. The LEC proliferation is restricted within the space of the remaining lens bag and does not approach the visual axis (101). However, it is cautioned that this prevents PCO only if the anterior and posterior capsules have been secured properly in the peripheral groove of the IOL (102).

**Polishing (scraping) the Anterior Capsule.** The overlap of the anterior capsule rim over the IOL optic leads to the EMT of the LECs, resulting in ACO. Several authors have evaluated the role of polishing the anterior capsule on the development of PCO when using silicone IOLs. Polishing of the anterior capsule has been effective in reducing fibrotic opacification but ineffective in reducing regeneratory opacification (103). In eyes with a sharp-edged silicone IOL, anterior capsule polishing caused no significant difference in fibrotic PCO (104).

### IOL Design

IOL design, optic size and edge, and its material are important considerations in retarding the development of PCO. Optics with biconvex designs retarded capsular opacification when there was a broad adhesion of the lens optic to the posterior capsule (105). A 6 mm IOL optic diameter was associated with less PCO than a 5.5 mm IOL optic (106).

Nishi *et al* suggest that IOLs with a sharp optic edge create an acute bend in the posterior capsule and this helps to a large extent in retarding the development of central PCO (107). The sharp capsule bend appears to represent a physical hindrance, which may induce contact inhibition of cell movement (108). A histopathological examination reveals that IOLs with a rounded edge show a capsular bend and complex folds in the posterior capsule that are not as sharp as that of a square-edged optic (107). Our clinical trials have also shown that the square-edged AcrySof IOLs led to significantly lower levels of PCO than the square-edged PMMA IOL at six months and one-year postoperatively (109). Based on the mathematical model, it has been predicted that IOLs with square-edged optic profiles exert higher pressure on the posterior capsule than IOLs with round-edged optic profiles. The higher pressure may form a physical barrier to prevent the migration of LECs onto the posterior capsule (110).

In addition to the optic edge, it has been observed that the haptic angulation also reduces the incidence of PCO by inducing a pressure gradient over the posterior capsule (111).

### IOL Material

It has been experimentally shown that in addition to the sharp rectangular optic edge, an IOL with an adhesive material promotes capsular adhesion. *In vitro* experiments have shown that a sharp discontinuous bend in the lens capsule is unlikely to be solely responsible for the observed reduction in posterior capsule opacification associated with the use of square-edged IOLs (112). In addition, the speed of the capsule bending depends on the material of the IOL (107). AcrySof material has a “tacky surface” with adhesive properties and experimental studies have revealed that collagen type IV, fibronectin and laminin adhered stronger and faster to the acrylate IOL (113). Sacu *et al* (114) noted that the capsule-IOL contact on optical coherence tomography was on the 10^th^ day with a single piece acrysof, on the 13^th^ day with a three-piece acrysof, and on the 15^th^ day with a three-piece silicone IOL.

**Figure 3:**
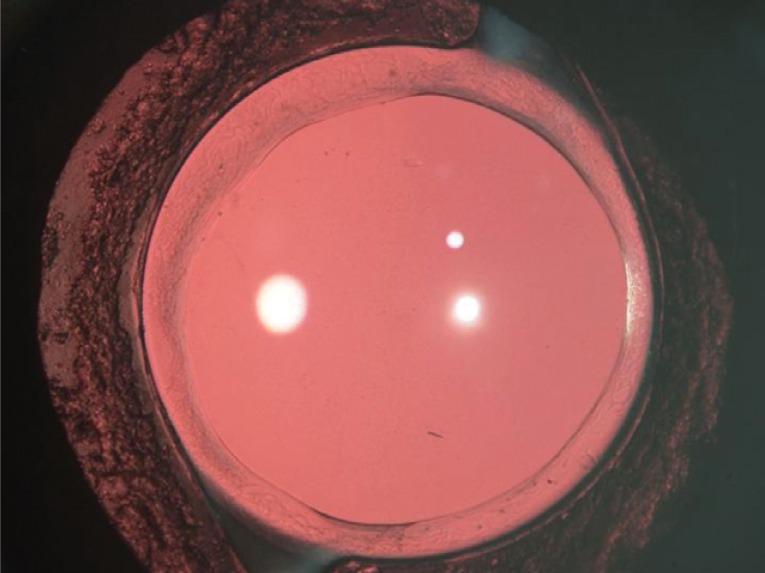
An eye at the 4 years follow-up with AcyrSof IOL implanted in the bag. There is no central PCO.

The incidence of PCO is the least following implantation of the AcrySof IOL (22) (Figure 3). A laboratory study has shown that the AcrySof IOL has a relatively low propensity to induce cellular proliferation in the capsular bag and has less Sommering’s ring formation than other IOL designs (115).

### Chemical Agents

A number of pharmacological agents have been proposed to prevent PCO, and most of these have been tested *in vitro*. These are anti-inflammatory, anti-proliferative, anti-adherence, anti-migrative, and anti-transdifferentiating in nature.

**Anti-inflammatory and Immuno-modulating Agents.** Anti-inflammatory and immuno-modulating drugs reduce the inflammatory response and secretion of cytokines, and this subsequently leads to a reduction in the proliferation of LECs. These drugs are indomethacin (116), diclofenac sodium (116) and cyclosporin A^117^. Topical instillation of diclofenac in the immediate post-operative period did not seem to influence the formation of PCO (118).

**Anti-proliferative Agents.** Anti-proliferative agents reduce the rate of proliferation of LECs and prevent the entry of LECs into the process, which leads to the development of PCO. The various anti-proliferative agents tested *in vitro* are 5-fluorouracil, mitomycin C (119), duanomycin (120), FGF receptor-1 antagonist SU5402 (58), octreotide (121), colchicines (122) and doxorubicin (123). The sustained release of mitomycin C suspended in sodium hyaluronate has shown to reduce PCO in rabbits (124). The proteosome inhibitor also leads to reduced proliferation of LECs in the *in vitro* condition (125). Application of electric fields has shown to decrease the entry of cells into the S-phase from the G1-phase by decreasing the G1-specific cell cycle protein cyclin E and increasing the cyclin-Cdk complex inhibitor p27kip1 (126).

**Anti-adhering and Anti-migratory Compounds.** Anti-adhering and anti-migrative compounds do not allow the attachment of LECs to the posterior capsule and thus prevent the migration of LECs to the posterior capsule. The various anti-migrating and anti-adherence substances tested are ilomastat (a matrix metalloproteinase inhibitor), naphtyl urea suramin (127), salmosin (a disintegrin) (128), mibefradil (Ca-channel inhibitor) (129), RGD peptide (38), EDTA (130), and coating an acrylic IOL surface with MPC polymer (131). They reduce proliferation of LECs by reducing prostaglandin E2 production by LECs.

**Cell Death Inducing Agents.** Anti-transdifferentiating agent minoxidil (lysyl hyroxylase inhibitor) (132) and liposome encapsulated TAH can inhibit metaplasia and proliferation of LECs (133). Inducing apoptosis of the residual LECs has been tried in experimental studies. The agents include bacteriochlorin A (134), fas ligand activating monoclonal antibody (135), ricin A (136), ricin A conjugated to an anti-LEC antibody (137), and 1% preservative free lidocaine (138). Adenovirus mediated Bax, procaspase 3 gene transfer, and distilled water have led to a reduction of PCO in the rabbit model (139). Indocyanine green and trypan blue have shown to kill LECs in a dose-dependent manner due to their photosensitization (140, 141).

### Delivery of Drugs

The drugs can be delivered within the capsular bag to the residual LECs by coating the IOL, modifying the irrigating medium, and during cortical cleaving hydrodissection, majority of which are still in the experimental phase.

**Coating the IOL.** The delivery via the IOL could provide a more controlled means of drug delivery that can concentrate an agent at the site of the potentially proliferating cells more efficiently. A heparin coated PMMA IOL reduces inflammation and the incidence of PCO (142). A thapsigargin (endoplasmic reticulum based Ca-ATPase inhibitor) coated IOL is also shown to reduce PCO (143). An indomethacin coated IOL also reduces mitosis (144). Behar-Cohen has reported the effects of an FGF-saporin complex bound to a heparin surface modified IOL in rabbits (145). A hydrophobic soft acrylic IOL coated with MPC polymer has shown to suppress adhesion and proliferation of LECs (131).

**Irradiation and Photodynamic.** Therapy Active oxygen processing by ultraviolet/ozone irradiation and argon plasma irradiation on the surface of Acrylic IOLs have been effective in preventing secondary PCO in 8-week-old albino rabbits (146). Photodynamic therapy with bacteriochlorin A (BCA) has induced lens epithelial cell death and greatly reduced the formation of a Soemmering’s ring (147). However, the corneas of the PDT-treated animals were opaque and swollen and had lost their endothelial lining (134).

**Irrigating Solution.** Low molecular weight heparin added to the irrigating fluid during cataract surgery has resulted in less fibrin and pigment deposits on the lens (148).

**Cortical Cleaving Hydro-dissection**. *In vitro* experiments showed that hydrodissection with 1% preservative-free lidocaine may help diminish the number of live LECs in the anterior capsule by facilitating cortical clean-up, by loosening the desmosomal area of cell-cell adhesion with decreased cellular adherence, or by a direct toxic effect (138).

Despite these *in vitro* and *in vivo* efforts these agents are yet not implicated for use in humans. With these drugs, there is a possibility of improper delivery or diffusion of these agents outside the capsular bag and this may adversely affect other surrounding tissues of the eye. The tissue most susceptible to these effects is the corneal endothelium (134). Other complications observed in the rabbit model are retinal necrosis and anterior chamber reaction (145). Therefore, the targeted delivery of these agents inside the capsular bag is important. Recently, a device, which can selectively apply certain agents to the residual LECs without harming other tissues of the eye, has been created (149).

## TREATMENT

**Figure 4:**
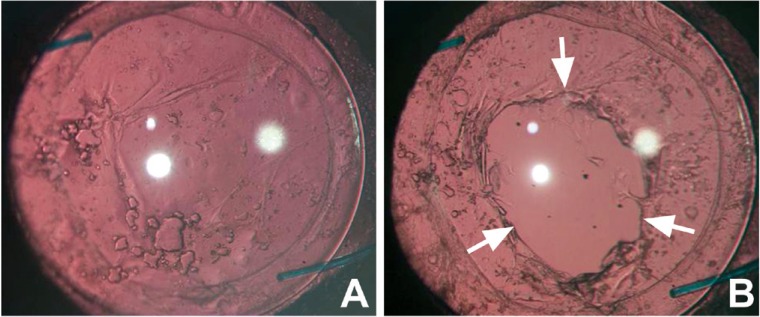
An eye with posterior capsule opacification before (A) and after Nd: YAG posterior capsulotomy (B).

Central PCO obscuring the visual axis can be treated with either surgical intervention such as posterior capsule scraping or with a non-surgical neodymium:YAG (Nd: YAG) laser capsulotomy. Figures 4A and 4B show an eye before and after Nd: YAG posterior capsulotomy. The latter method has received wide acceptance. Performing Nd: YAG capsulotomy of an appropriate size and at an appropriate site is challenging. A capsulotomy that is larger than the pupil diameter under scotopic conditions may prevent disturbances of vision such as monocular diplopia (150). The clinical complications from Nd: YAG laser capsulotomy includes a rise in intraocular pressure, glaucoma, cystoid macular edema, and retinal detachment (151). Therefore, it is important that strategies to retard and prevent PCO may contribute to preserving visual acuity in patients over their lifetimes.

## PCO IN CHILDREN

Children display a significantly higher incidence of postoperative PCO and a higher Nd: YAG laser posterior capsulotomy rate (152). In children, the re-operation rate following congenital cataract surgery is significantly higher than in age-related cataracts and especially higher with an intact posterior capsule (153). An *in vitro* experiment of human capsules with a central hole of 5 mm (PCCC) placed in a culture showed that even in the absence of the posterior capsule, their natural substrate, LECs that remain after cataract extraction have the potential to proliferate and form a monolayer of LECs on a basal lamina of vitreous origin. These LECs are able to close the posterior capsulorrhexis partially or totally in approximately one-third of cases (154).

### Surgical Prevention

In children, multiquadrant cortical cleaving hydrodissection ensures thorough, complete, easy, and rapid removal of the nucleus in eyes with an intact capsule (155). Primary posterior continuous curvilinear capsulorhexis may delay the onset of central PCO and primary anterior vitrectomy may be necessary to prevent or eliminate the onset of central PCO (155, 156). In pediatric eyes, AcrySof IOL implantation with appropriate management of the posterior capsule helps in retarding the development of central PCO maintaining a clear visual axis in a majority of the cases (155, 157). The PCO profile following AcrySof IOL implantation was different from that of PMMA IOL implantation. With PMMA, the opacification sets in rapidly in the first few weeks. The type of PCO seen with PMMA is fibrous. It is fierce, not only in its appearance but also in its impact on the development of amblyopia. In contrast, following Acrysof implantation, the type of PCO is predominantly proliferative. PCO sets in at a later stage, typically at 14-16 months. Visual obstruction produced with Acrysof IOL is less severe than with PMMA IOL and is therefore less amblyogenic (155). Grieshaber *et al* have suggested anterior and posterior vertical capsulotomy, with optic entrapment of the intraocular lens to avoid vitrectomy in infants under the age of 5 years. The fusion of the anterior and posterior capsules in front of the IOL limits the proliferation and migration of Elschnig pearls (158).

### Treatment

In younger children, visual axis obscuration due to PCO can be treated with pars plana vitrectomy and membranectomy. In older children and adults an Nd: Yag posterior capsulotomy is an acceptable option for the management of PCO (159). Retinal detachment after Nd: YAG capsulotomy for PCO is rare in eyes that had previous uneventful phacoemulsification and PC IOL implantation (160). A second Nd: YAG posterior capsulotomy that could be required can also be carried out. Recently a transconjunctival sutureless vitrectomy system has been employed (161).

## CONCLUSION

In conclusion, capsular opacification, in particular, PCO, still remains a physiological complication of an uneventful cataract surgery. The quest for its eradication goes on. Different patient-related factors, ocular and systemic conditions, surgical techniques to remove the residual LECs, and residual cortical fibers and adjuncts influence the development of PCO. The use of intraocular lenses with different biomaterials and edge designs can influence the progression of visually significant PCO. At present, meticulous use of surgical techniques and appropriate intraocular lens remain the mainstays for retarding the development of post-operative posterior capsule opacification in humans.
